# Frequency of bullying modulates amygdala response during social exclusion in participants with non-suicidal self-injury

**DOI:** 10.1007/s00787-025-02940-1

**Published:** 2026-01-16

**Authors:** Jennifer Spohrs, Birgit Abler, Andreas G. Chioccheti, Ulrich W. Ebner-Priemer, Jörg M. Fegert, Saskia Höper, Tina In-Albon, Michael Kaess, Michael Koelch, Elisa Koenig, Julian Koenig, Laura Kraus, Anna Michelsen, Inga Niedtfeld, Paul L. Plener, Sandra Nickel, Philip Santangelo, Cedric Sachser, Christian Schmahl, Maurizio Sicorello, Elisa Sittenberger, Patrice van der Venne, Andreas Witt, Georg Grön, Martin Ulrich, the STAR Consortium, Kathrin Brändle, Lisa Schischke, Alina Geprägs, Isabell Liebhart, Rebecca Brown, Vera Münch, Elisa König, Ulrike Hoffmann, Jenny Zähringer, Hasan-Hüseyin Isik, Olaf Reis, Silvia Lindlar, Regina Waltes, Markus Mössner, Elisa Flach, Alexandra Edinger, Franziska Binder, Stephanie Bauer, Margarete Mattern, Sabine Herpertz

**Affiliations:** 1 Department for Child and Adolescent Psychiatry and Psychotherapy, Ulm University Medical Centre, Ulm, Germany; 2 Department of Psychiatry, Psychotherapy and Psychotraumatology, Military Medical Centre, Ulm, Germany; 3 Department of Psychiatry and Psychotherapy III, Section Neuropsychology and Functional Neuroimaging, Ulm University Medical Centre, Ulm, Germany; 4 Department of Child and Adolescent Psychiatry, Psychosomatics and Psychotherapy, Goethe University Frankfurt, Frankfurt am Main, Germany; 5 Mental mHealth Lab, Institute of Sports and Sports Science, Karlsruhe Institute of Technology (KIT), Karlsruhe, Germany; 6 Department of Psychiatry and Psychotherapy, Medical Faculty Mannheim, Central Institute of Mental Health, Heidelberg University, Mannheim, Germany; 7 German Centre for Mental Health (DZPG), partner site Mannheim, Heidelberg, Ulm, Germany; 8 German Centre for Child and Adolescent Health (DZKJ), partner site Rostock, Ulm, Germany; 9 Department for Child and Adolescent Psychiatry, Centre of Psychosocial Medicine, University Hospital Heidelberg, Heidelberg, Germany; 10 Clinical Child and Adolescent Psychology and Psychotherapy, University of Mannheim, Mannheim, Germany; 11 University Hospital of Child and Adolescent Psychiatry and Psychotherapy, University of Bern, Bern, Switzerland; 12 Department for Child and Adolescent Psychiatry and Psychotherapy, Rostock University Medical Centre, Rostock, Germany; 13 Faculty of Medicine, Department of Child and Adolescent Psychiatry, Psychosomatics and Psychotherapy, University Hospital Cologne, University of Cologne, Cologne, Germany; 14 Department of Psychosomatic Medicine and Psychotherapy, Central Institute of Mental Health Mannheim, Medical Faculty Mannheim, Heidelberg University, Mannheim, Germany; 15 Department of Child and Adolescent Psychiatry, Medical University of Vienna, Vienna, Austria; 16 Department of Behavioural and Cognitive Sciences, University of Luxembourg, Esch-sur-Alzette, Luxembourg; 17 Department of Clinical Child and Adolescent Psychology, Institute of Psychology, University of Bamberg, Bamberg, Germany

**Keywords:** Non-suicidal self-injury, Cyberball, Social exclusion, Bullying, FMRI, Perfusion imaging

## Abstract

Non-suicidal self-injury (NSSI) is a phenomenon of high worldwide prevalence. Social exclusion as a major form of bullying has been reported to be an important risk factor for NSSI. We investigated how different frequencies of previous bullying experiences affect the neural processing of social exclusion in groups of female adolescents and young adults engaging in NSSI (*n* = 57), and matched healthy controls (*n* = 58). The Cyberball paradigm was used to experimentally contrast the neural effects of social exclusion relative to social inclusion. MR-based perfusion imaging measuring changes in regional cerebral blood flow (rCBF) was served as a marker of functional brain activation. Without considering previous bullying experiences, group comparisons revealed that individuals engaging in NSSI showed greater differential activation of lateral temporal brain regions, anterior hippocampus, and amygdala during social exclusion compared to healthy controls. The frequency of past bullying experiences modulated this activation pattern. Increased differential activation was particularly driven by NSSI individuals with occasional bullying experiences (around 0.5–1 time per month) while NSSI individuals with a higher frequency showed the lowest magnitude of the “exclusion > inclusion” effect. The blunted amygdala activation of NSSI individuals with a higher frequency of bullying experiences could be interpreted as a sign of emotional numbing. Following this putative interpretation, the results suggest that emotional numbing should be given the same attention as increased sensitisation to social exclusion during the treatment of NSSI to promote functional emotion regulation.

**Supplementary Information:**

The online version contains supplementary material available at 10.1007/s00787-025-02940-1.

## Introduction

 Non-suicidal self-injury (NSSI), defined as intentional, self-directed damage of body tissue without suicidal intent, has been described as a distinct clinical phenomenon [1]. Worldwide prevalence is high, with 25–33% of adolescents deliberately hurting themselves at least once [2–4] and around 4% repetitively [5, 6]. The highest prevalence has been found around the age of 15–16 years [7]. Frequent mental disorders associated with NSSI comprise borderline personality disorder (BPD), depression, anxiety disorders, post-traumatic stress disorder (PTSD), substance use disorders, and eating disorders, as well as an increased risk for suicidal behaviour [8–10]. A multifactorial model with interdependent biological, psychological, and social factors has been proposed to explain the development and maintenance of NSSI [11]. Furthermore, by generating strong sensory experiences (e.g., pain [12, 13]), NSSI is considered as a means to end states of dissociation, depersonalisation, and emotional numbing, defined as a limited capacity to experience and process intense emotions [14].

The experience of social exclusion has been reported as a major risk factor promoting NSSI [3, 15, 16], and social exclusion represents one of the major forms of bullying with generally negative consequences on mental health [17–22] and NSSI specifically [23, 24]. In young adults engaging in NSSI [25] and adolescents who have experienced bullying [26], neural processing of social exclusion was found to be altered.

The “Cyberball” paradigm is an established way to experimentally investigate the neural correlates of social exclusion [27]. It is a game with three different conditions, in which participants are instructed to observe, or to participate in a ball tossing game. During the two participation conditions, the subject is either fully included in ball tossing or becomes excluded after an initial phase of inclusion. Both conditions reliably lead to feelings of either social inclusion or exclusion. Contrasting neural activation during exclusion against inclusion in young healthy adults [28–31] yielded greater activation of salience coding brain regions: the anterior insular cortex, the ventral lateral prefrontal cortex (vlPFC), and the medial PFC (mPFC). Considering previous experiences of bullying in healthy adolescents, Kiefer et al. (2021) [32] reported a positive relationship between the increase in neural signalling related to social exclusion and prior bullying experiences. These associations were most prominent for the left inferior frontal cortex and the subgenual anterior cingulate cortex. Investigating the neural signature for social exclusion in youths committing NSSI, Groschwitz et al. (2016) [25] found enhanced activation of the mPFC and the vlPFC in depressed adolescents with NSSI as compared to depressed adolescents without NSSI. However, this study did not address if and how previous bullying experiences would modulate brain activation during social exclusion in participants with NSSI.

Therefore, the aim of the present study was to investigate the relation between the neural correlates of social exclusion and individual experiences of bullying in larger samples of adolescents with NSSI and healthy controls (HC) using magnetic resonance imaging (MRI)-based perfusion imaging, very similar to the neuroimaging method used in Kiefer et al. (2021) [32]. We expected that, relative to controls, participants with NSSI would generally show altered activation of different salience coding brain regions during social exclusion relative to inclusion [25, 33, 34]. We further explored whether the frequency of previous bullying experiences would modulate brain activation patterns in participants with NSSI. We used the Olweus Bully/Victim Questionnaire (OBVQ [35, 36]) to classify the participants with NSSI based on the self-reported frequency of bullying in the months prior to the study, resulting in three NSSI subgroups with different frequencies of past bullying (none, occasional, frequent). Conjoint pairwise comparisons between these subgroups were used to infer significant group differences, employing a statistically more conservative implementation of polynomial trend tests across subgroups.

## Materials and methods

This study is a subproject of the STAR (*S*elf-Injury: *T*reatment *A*ssessment *R*ecovery) project, with general methodology and participant recruitment details provided in Spohrs et al. (2024) [37]. The study has been preregistered at Open Science Framework, OSF (https://osf.io/fwyjd). The study was conducted according to the guidelines of the Declaration of Helsinki and approved by the respective Ethics Committees of the participating study centres. All participants signed an informed consent before being included.

### Participants

Participants were recruited through multiple channels, including personal communication, social media, contact in the context of patient’s presentation at the hospital for treatment, and presentations at local schools.

Of the initially recruited 66 female adolescents with NSSI and 60 female age-matched healthy controls, five participants withdrew from the study during MRI scanning. The data of four participants were excluded from analysis due to excessive movement during MRI scanning (frame-wise displacements > 4 mm). Data from the OBVQ were missing for another two participants, leading to final samples of 57 participants with NSSI (mean age = 19.6 years, SD = 2.1 years) and 58 healthy controls (mean age = 19.5 years, SD = 2.3 years).

### Experimental task during perfusion imaging

We employed the “Cyberball” task [27, 38, 39], a validated virtual ball-tossing paradigm designed to simulate scenarios of social inclusion and exclusion. Participants were led to believe they were engaging in a ball-tossing game with two other individuals. However, the gameplay was entirely pre-programmed, with no actual human players involved. The participants’ avatar, depicted as a hand at the screen’s bottom, interacted with animated figures representing the other “players” (Figure S1). After the experiment, participants were debriefed regarding the task’s deceptive aspects and the game’s controlled nature to address ethical considerations.

The task encompassed three distinct conditions, each lasting about 2 min, featuring around 60 ball tosses: passive watching (P), inclusion (I), and exclusion (E), each executed three times, in the following pseudorandomised sequence: P-I-E-I-E-P-E-P-I. During the passive watching condition, participants observed the game without direct involvement. In the inclusion condition, participants randomly received the ball in one third of all ball throws and were instructed to toss the ball to the other players, allowing for active participation in the game. Conversely, the exclusion condition began with a brief inclusion phase for the first 20 s, but thereafter only the virtual players tossed the ball to each other, thereby operationalising social exclusion. The experiment was controlled by Presentation 18.1 (Neurobehavioral Systems Inc., San Francisco, USA). The visual components of the experiment were displayed on a liquid crystal display (LCD) screen (NordicNeuroLab AS, Bergen, Norway) positioned behind the scanner. Participants viewed these stimuli through a mirror attached to the MRI head coil.

### Psychometric measurements

After MR scanning, participants completed the revised Olweus Bully/Victim Questionnaire [35, 36] in German language. For this study, the global item “How often have you been bullied at school in the past couple of months?” was selected to classify participants with NSSI into three subgroups: NSSI participants with no bullying experience in the last couple of months (“none” group), NSSI participants subjected to bullying 0.5–1 time per month (“occasional” group), and NSSI participants bullied twice per month or more (“frequent” group). Categorial cutoffs were used for two reasons: the variable did not exhibit sufficient continuity, and the categorial approach is clinically more familiar, informative and interpretable.

Additionally, to further characterise participants’ clinical profiles, the following instruments were administered (in German language): the Borderline Symptom List-23 (BSL-23) [40], the Childhood Trauma Questionnaire (CTQ) [41], NSSI severity (NSSV-SG) [42], and the Patient Health Questionnaire-9 for Adolescents (PHQ-9 A) [43].

### MRI data acquisition

MRI data were acquired using Siemens 3-Tesla scanners (Erlangen, Germany), specifically a MAGNETOM Prisma at the Department of Psychiatry of Ulm University, and a MAGNETOM Prisma^fit^ at the Department of Psychosomatic Medicine and Psychotherapy, Central Institute of Mental Health Mannheim, both equipped with standard 64-channels head/neck coils (Siemens, Erlangen, Germany). Participants were positioned inside the MR scanners, with their heads securely cushioned to reduce movement-related artefacts during data acquisition. To index energy-intensive neuronal activation during the Cyberball task, regional cerebral blood flow (CBF) was measured using pseudo-continuous arterial spin-labelling. The 3D gradient-echo spin-echo imaging sequence was developed at the German Centre for Neurodegenerative Diseases (DZNE), Bonn, Germany. In line with the recommendations by Alsop et al. (2015) [44] for MRI-based perfusion imaging, the following parameters were used: repetition time (TR): 4000 (2 TRs per volume), echo time (TE): 24 ms, matrix size: 64 × 64, field-of-view (FOV): 240 mm, 34 slices, slice thickness: 4 mm (no gap), transverse slice positioning along the AC-PC line, ascending slice acquisition, flip angle: 90°/120° (slab-selective), PAT factor: 2 (GRAPPA mode), bandwidth: 2232 Hz/pixel, marking duration (bolus length): 2400 ms, transit time (post-labelling delay): 1000 ms, pre-saturation and background suppression (BS) switched on, BS method: 2 pulses, voxel size: 3.75 mm × 3.75 mm × 4.00 mm. The duration of each perfusion sequence was set to 125 s, capturing six label and six control images, along with two M0 (proton-density weighted) images before the commencement of perfusion scanning. After the Cyberball task, a high-resolution T1-weighted anatomical image was acquired using a 3D magnetisation prepared rapid acquisition gradient echo sequence (MPRAGE).

### Neuroimaging data analysis

The preprocessing and statistical analyses of the imaging data were performed using Statistical Parametric Mapping (SPM12 r6225, Wellcome Department of Cognitive Neurology, London, UK) running on MATLAB 2021a (MathWorks Inc., Natick, MA, USA).

After confirming no significant influence of scanning site on the effects of interest (see Supporting Information), we proceeded to enter the condition-specific contrast images into a second-level analysis (flexible factorial design) in SPM12. The model featured a between-subjects factor, “Group”, with four levels arranged in this order: Group 1: Healthy controls; Group 2: NSSI participants with no bullying experience; Group 3: NSSI participants, bullied 0.5–1 time per month; Group 4: NSSI participants, bullied ≥ 2 times per month. Furthermore, the within-subjects factor “Condition”, with two levels (“social inclusion” and “social exclusion”), was added to the model, as well as a factor “Subjects”, to account for interindividual variance in estimated neural activation during each task condition against baseline.

After model estimation, the contrast “social exclusion > inclusion” was computed separately for the entire NSSI group (averaged across subgroups) and healthy controls (see Figure S2 for results). Next, we investigated differences in the “social exclusion > inclusion” effect between the entire NSSI group and healthy controls. Two one-sided t-contrasts, testing the directions “NSSI > HC” and “HC > NSSI”, were computed at the whole-brain level by applying a voxel-height threshold of *p* < 0.001. To achieve a family-wise error rate (FWE)-correction (*p* < 0.05) at the cluster level, a cluster extent threshold of k = 343 contiguously significant voxels was employed.

Confined to significant clusters from the above analysis, we then investigated the potential modulating influence of past bullying experiences on the “social exclusion minus inclusion” activation difference among the three NSSI subgroups (excluding healthy controls). Given the arrangement of NSSI subgroups along the dimension bullying frequency with levels „none“, „occasional“ and „frequent“, trend tests had been the appropriate method to test for linear and quadratic between-group differences. However, trend tests do not inform about the significance of between-group differences, which is why we used conjoint pairwise group comparisons as an alternative method. For instance, for a linear increase, we tested whether the “social exclusion > inclusion” difference in neural activation was significantly higher for the “occasional” group than the “none” group ([-1 1 0]) and whether conjointly the “frequent” group would show higher differential activation when compared against the “occasional” group ([0-1 1]). This conjoint between-group testing can only be significant in case of parametrically increasing differential neural activation across groups. Given the directionality of the conjoint between-groups testing, the same procedure was used for parametrically decreasing differential neural activation ([1-1 0] AND [0 1-1]). Similarly, as an alternative for quadratic trend tests, conjoint pairwise group comparisons asked for significantly greater differential neural activation of the “occasional” group compared to the “none” group ([-1 1 0]) and the “frequent” group ([0 1-1]). Again, contrast directions were inverted for both directed t-tests to ask whether the “occasional” group exhibited significantly less differential neural activation compared to the “none” and “frequent” groups ([1-1 0] AND [0- 1 1]). Each conjunction of contrasts was assessed at a voxel-height threshold of *p* < 0.05, in combination with a cluster extent threshold requiring at least 10 contiguously significant voxels.

## Results

### Psychometric measurements

Table S1 shows the mean age, and mean scores derived from the CTQ, PHQ-9 A, and BSL-23 for the healthy control group and the entire group of participants engaging in NSSI, together with statistical values obtained from two-sample t-tests. Both groups differed significantly in all psychometric measurements.

When comparing the different NSSI subgroups according to the frequency of bullying experiences for the same set of variables and including NSSI severity, one-way analyses of variance with factor “group” did not reveal any significant effects of this factor. Data are summarised in Table 1.

**Table 1:** Means ± standard deviations of demographic and psychometric measurements for each of the three participant subgroups engaging in non-suicidal self-injury (NSSI), accompanied by F-test values and associated p values derived from analyses of variance. The number of participants differs across variables due to missing data. Abbreviations: BSL-23: Borderline Symptom List-23; CTQ: Childhood Trauma Questionnaire; PHQ-9 A: Patient Health Questionnaire-9 for adolescents

Variable	NSSI, no bullying experience	NSSI, bullied ≤ 1× per month	NSSI, bullied ≥ 2× per month	F value	*p* value
Age	19.6 ± 2.1 (*n* = 29)	19.6 ± 1.1 (*n* = 13)	19.6 ± 2.6 (*n* = 15)	F(3, 111) = 0.02	0.996
CTQ, Emotional Abuse	14.6 ± 5.5 (*n* = 24)	16.5 ± 4.6 (*n* = 12)	18.3 ± 5.6 (*n* = 12)	F(2, 45) = 2.06	0.139
CTQ, Physical Abuse	6.8 ± 2.3 (*n* = 24)	7.3 ± 3.0 (*n* = 12)	9.3 ± 4.7 (*n* = 12)	F(2, 45) = 2.60	0.085
CTQ, Sexual Abuse	7.5 ± 4.6 (*n* = 24)	9.2 ± 3.8 (*n* = 12)	10.1 ± 6.7 (*n* = 12)	F(2, 45) = 1.17	0.321
CTQ, Emotional Neglect	15.1 ± 5.8 (*n* = 24)	15.3 ± 4.3 (*n* = 12)	15.5 ± 4.5 (*n* = 12)	F(2, 45) = 0.03	0.972
CTQ, Physical Neglect	9.3 ± 4.6 (*n* = 24)	9.8 ± 3.6 (*n* = 12)	11.3 ± 5.0 (*n* = 12)	F(2, 45) = 0.86	0.430
PHQ-9 A	17.1 ± 6.2 (*n* = 27)	15.8 ± 5.6 (*n* = 12)	18.3 ± 5.0 (*n* = 13)	F(2, 49) = 0.57	0.570
BSL-23	46.9 ± 20.8 (*n* = 27)	49.2 ± 18.5 (*n* = 12)	58.1 ± 18.7 (*n* = 13)	F(2, 49) = 1.42	0.252
NSSI severity	5.1 ± 0.8 (*n* = 24)	5.0 ± 0.9 (*n* = 12)	5.2 ± 0.7 (*n* = 13)	F(2, 46) = 0.12	0.891

### Neuroimaging data

**Fig. 1:**
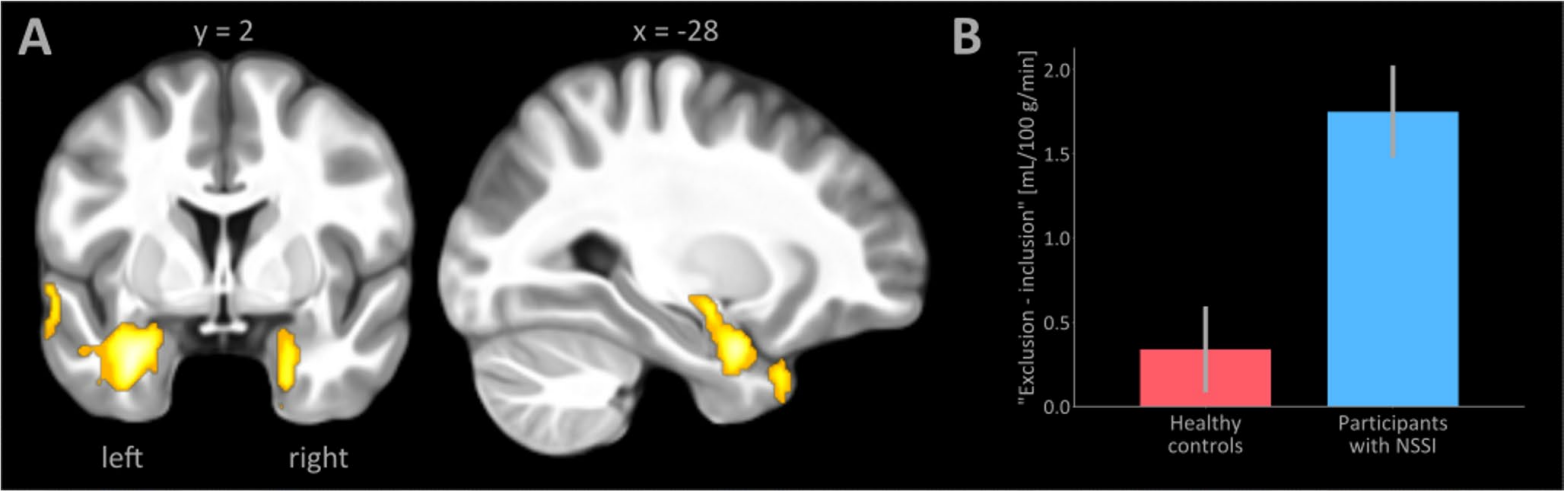
(**A**) Brain regions significantly more activated during social exclusion compared to inclusion in participants with non-suicidal self-injury (NSSI; *n* = 57), relative to healthy controls (*n* = 58). The statistical parametric map was thresholded at *p* < 0.001, family-wise error rate-corrected (*p* < 0.05) at the cluster level and overlaid onto the mean normalised skull-stripped T1 image (averaged across all 115 participants), using MRIcroGL [45]. Coordinates are in Montreal Neurological Institute space. (**B**) Bar chart showing the differential (“exclusion minus inclusion”) mean regional cerebral blood flow (rCBF; mL/100 g/min) for both groups, averaged across all voxels within the left amygdala using an anatomical mask (obtained from the SPM Anatomy Toolbox 3.0 [46–48]. Error bars denote the standard error of the mean

We tested for a greater effect of social exclusion minus inclusion in individuals with NSSI relative to healthy controls and identified two significant clusters (Table 2; Fig. 1). The first cluster was located in the left hemisphere and comprised 1550 voxels, with its global peak voxel in the left amygdala. This cluster extended into adjacent areas of the anterior hippocampus, the temporal pole, and the superior and middle temporal gyri. A homologous but smaller cluster in the right hemisphere, consisting of 779 voxels, included parts of the amygdala and the temporal pole. No significant results were obtained for the opposite contrast, asking for greater activation for exclusion than inclusion in healthy controls compared with NSSI participants.

**Table 2:** Brain regions exhibiting significantly higher activation during social exclusion compared to inclusion in participants with NSSI (*n* = 57), relative to healthy controls (*n* = 58). A voxel-height threshold of *p* < 0.001 and family-wise error rate correction (*p* < 0.05) at the cluster level were applied. Coordinates are in Montreal Neurological Institute (MNI) space

Brain region	Number of voxels	Peak voxel (MNI space)			
		x	y	z	z-score
Left amygdala	1550	−32	4	−28	4.72
Left superior temporal gyrus		−60	0	−8	4.45
Left hippocampus		−32	−6	−16	3.89
Left middle temporal pole		−32	20	−34	3.81
Left superior temporal pole		−50	20	−24	3.76
Left middle temporal gyrus		−58	6	−18	3.59
Right superior temporal pole	779	46	18	−24	4.41
Right amygdala		24	4	−28	3.81
Right middle temporal pole		32	22	−34	3.59

**Fig. 2:**
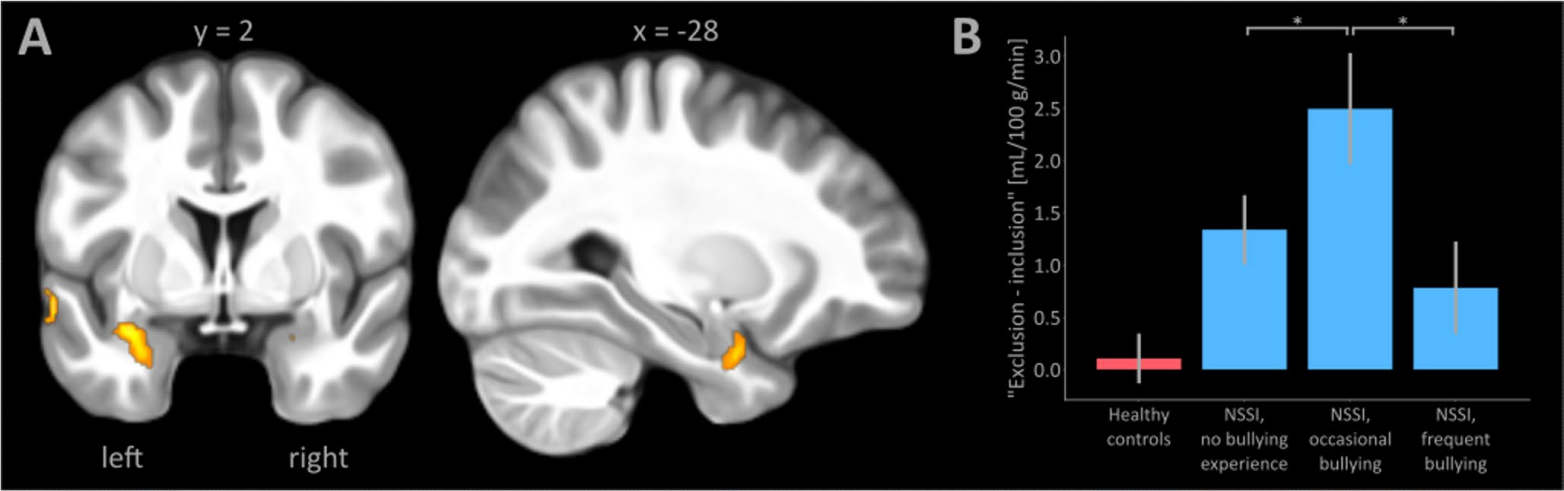
(**A**) Brain regions whose differential activity (“social exclusion >inclusion”) demonstrated an inverted U-shaped pattern for the frequency of bullying experiences among the three subgroups with non-suicidal self-injury (NSSI). The conjunction of the two underlying contrasts was evaluated using a voxel-height threshold of *p* < 0.05 and a minimum cluster size of 10 voxels. Coordinates are in MNI space. (**B**) Bar chart depicting the “social exclusion minus inclusion” difference in mean regional cerebral blood flow (rCBF; mL/100 g/min) for the four participant groups. The difference between the “frequent” and “no bullying experiences” group was significant (*p* < 0.05), too. Differential rCBF was extracted and averaged across all significant voxels within the left amygdala, as defined by the SPM Anatomy Toolbox 3.0 [46–48]. Error bars denote the standard error of the mean. ^*^ *p* < 0.05. Please note that data for healthy controls (red bar) are shown for comparison but were not included in any of the statistical analyses leading to the results presented here

Exploring the frequency effect of bullying experiences by conjoint pairwise comparisons between NSSI subgroups yielded results summarised in Table 3; Fig. 2.

The most substantial “exclusion > inclusion” effect was observed for the “occasional” group (Fig. 2B). The exclusion effect was significantly greater in this group compared to both the group with no bullying experience and the group that frequently experienced bullying, which showed the lowest differential “exclusion > inclusion” effect. That group’s effect differed significantly not only from the “occasional” group (see above), but also from the group with no previous bullying experience (“none”) as revealed by an additional t-contrast. No other conjunction analyses of pairwise group comparisons showed significant results, supporting the specificity of the observed results pattern (Fig. 2B; for reasons of brevity, we refer to this pattern as “inverted U-shaped” throughout the manuscript, although it is based on a categorical between-group contrast rather than a statistically modelled trend analysis, such as a quadratic regression).

**Table 3:** Brain regions where the “social exclusion > inclusion” effect varied across the three NSSI subgroups as a function of bullying frequency, following an inverted U-shaped pattern (see also Fig. 2B). The two underlying defining contrasts were conjoined (see Methods), using a voxel-height threshold of *p* < 0.05 and a cluster extent threshold of k = 10 voxels. Coordinates are in Montreal Neurological Institute (MNI) space

Brain region	Number of voxels	Peak voxel (MNI space)			
		x	y	z	z-score
Left superior temporal gyrus	32	−60	2	−10	2.78
Left hippocampus	269	−34	−8	−20	2.31
Left amygdala		−34	2	−18	2.31
Right amygdala	66	24	4	−22	1.81

## Discussion

The present study investigated the modulating effect of previous bullying experiences on neural activation during social exclusion in a large sample of young adults and adolescents engaging in non-suicidal self-injury (NSSI), and a healthy control (HC) group, using MR-based perfusion imaging. For experimental realisation, the ball-tossing game “Cyberball” [27, 38] was implemented. Comparing the neural activation effect of social exclusion vs. inclusion between the NSSI group and the HC group, without considering the putative influence of previous bullying experiences, we identified significant group differences in left lateral temporal regions, comprising the temporal pole and superior and middle temporal gyri. Medially, the left anterior hippocampus, and bilateral amygdala showed significant group differences. These results deviate from the expectations formulated in the preregistration. However, the predictions there were based on the results of earlier studies that had used a different imaging method with blood oxygen level dependent (BOLD)-fMRI only, whereas in the current study MR-based perfusion imaging was used in combination with a markedly larger number of repeated and longer lasting experimental conditions.

When exploring the result pattern for the frequency effect of previous bullying experiences, it became evident that this effect was particularly driven by individuals with NSSI who had experienced bullying “occasionally” (0.5–1 time per month), and by the NSSI group with two or more bullying experiences per month (“frequent”) presenting the lowest magnitude of the “exclusion > inclusion” effect.

As none of the other clinical variables (Table 1) showed reliable differences between NSSI subgroups, it is most likely that the frequency of previous bullying experiences was the primary driver of the inverted U-shaped activation observed in the amygdala.

The overall results pattern suggests that previous bullying experiences modulate the neural response to social exclusion in NSSI patients differentially. While occasional bullying experiences appear to sensitise NSSI subjects increasing their amygdala response, the blunted differential amygdala activation of the NSSI group with more frequent bullying experiences could be interpreted to indicate emotional numbing towards acute social exclusion.

### Social exclusion effects without considering previous bullying experiences

The greater left amygdala activation in response to social exclusion relative to inclusion across all NSSI subgroups vs. HC indicates that the NSSI subjects presented, on average, increased relative arousal to the aversive situation of social exclusion, which aligns with previous studies investigating the neural underpinnings of aversive stimulus processing in young adults with NSSI [49, 50]. In line with models of NSSI, suggesting amplified emotional responding as a core symptom of NSSI [51, 52], the relative increase in amygdala activation could reflect its underlying neural mechanism. However, a comparison with previous results is also challenging since those previous studies have contrasted samples with either comorbid depression or borderline personality disorder (BPD) [25, 53]. Furthermore, amygdala activation has rarely been reported by previous studies using the Cyberball paradigm, which could be due to the smaller sample sizes and different sampling methods to assess neural activation (e.g., BOLD functional MRI). However, in a larger sample (*n* = 45) using the Cyberball paradigm, heightened amygdala activation and peer victimisation have been reported as being associated [54].

Our findings further extend the literature by presenting altered activation of the temporal pole and superior and middle temporal gyri in the NSSI group compared to HC. Previously, Schreiner et al. (2017) [55] observed altered resting-state functional connectivity between these regions and the amygdala in youths engaging in NSSI relative to HC. Earlier findings have identified the temporal lobe to be involved in explicit emotional memories [56, 57], partially in suicidal thoughts and behaviour [58], and the temporal pole has been proposed to couple perceptual inputs to emotional responses, crucial for social behaviour [59]. As such, the temporal lobe could be regarded as a typical area processing social cues. Additionally, the temporal pole plays a greater role in theory of mind models. Previous functional MRI studies identified the temporal pole to be activated when participants were required to infer the mental states of others [60, 61], which fits the increased activation during the exclusion condition, where participants might have begun to consider the motives behind being excluded by the other players. Elucidating the role of the temporal pole in processes of social exclusion may be an avenue for future research.

### Social exclusion effects considering previous bullying experiences

Considering the frequency of past bullying experiences, an inverted U-shaped pattern of brain activation emerged. Social exclusion elicited highest amygdala activation in the NSSI group that had been less frequently confronted with bullying (“occasional”; 0.5–1 time per month). This increased activation of the amygdala associated with social exclusion is consistent with other findings [33, 49, 54], and previous studies have found neural correlates of social exclusion to be heightened in youths affected by peer victimisation [54, 62, 63]. Insofar, present results align with suggestions that this group is most sensitised and vulnerable to social exclusion, which reflects a potential risk factor for the maintenance of NSSI behaviour and should be accounted for in treating those individuals engaging in NSSI.

In contrast, NSSI individuals most affected by bullying (“frequent”; ≥ 2 times per month) showed the lowest social exclusion-related amygdala activation, which can be interpreted as a neural marker of “emotional numbing” [14]. Defined as a limited capacity to experience intense emotions, it is not implausible to assume that this state results from the more frequent exposure to bullying in the past. As one putative mechanism, chronic stress-associated over-activation of the hypothalamic-pituitary-adrenal (HPA) axis may play a role, resulting in persistently elevated levels of cortisol that could impair the integrity of the anterior hippocampus and modulate the structure and function of the amygdala [64, 65]. Future studies could integrate measurements of cortisol (serum, saliva, or hair) or the cortisol awakening response to estimate acute and chronic HPA axis activity to lend further support for that putative mechanism [66–68]. Serial measurement would even permit tracking of HPA axis dysregulation over time, which when integrated with psychometric and clinical assessments could help monitor the impact of interventions [69].

Support for our interpretation also comes from other research domains. For instance, PTSD has been linked to both hyper-responsivity to negative stimuli and emotional numbing [14, 70, 71]. Even though PTSD and NSSI are two distinct clinical phenomena, both share stressful and/or traumatic events, and disturbed emotional processing and regulation in their etiologic models [11, 72–74]. It is noteworthy that there is a growing discussion whether bullying can be understood as traumatic event, increasing the likelihood for developing PTSD [75, 76]. Emotional numbing is also coherent with models for both phenomena, since the reduced capability to initiate a prompt and appropriate emotional response to an aversive situation may in turn lead to hindered emotion regulation [77]. In addition, Sippel et al. (2018) [73] have identified affected social connectedness related to emotional numbing in patients with PTSD. Further highlighting the importance of emotional numbing, Seitz et al. (2024) [78] identified trait dissociation as an important confounder between childhood maltreatment and amygdala response to threat, and Sicorello et al. (2021) [79] have found that adverse childhood experiences during particularly sensitive periods of life are linked to reduced amygdala reactivity when viewing threatening images.

Emotional numbing has also been investigated in pain studies [80, 81]. For example, Korem et al. (2022) [81] found that emotional numbing was negatively correlated with amygdala reactivity to mild pain in patients with PTSD. Given that the amygdala is involved in processing both physical and emotional pain [82], and since emotional numbing has been found to affect both processes [72, 83], it is noteworthy that social rejection activates similar brain regions as those observed in physical pain studies [38].

### Limitations

When interpreting the results, some limitations need to be considered. Even though we present data from a large sample, participants were females only, potentially limiting the generalisability of the results. Future studies should incorporate male participants to allow a direct comparison between bullied females and males engaging in NSSI. Furthermore, participants were predominantly of white Caucasian ethnicity, residing in a mix of urban and rural areas in South-West Germany, which limits the generalisability of present findings. Generalisability is further constrained by the relatively small sample sizes within each bullying category. Group categorisation was based on bullying frequency assessed via a single-item global self-report, which may be subject to recall bias. Finally, we did not employ a specific questionnaire to assess emotional numbing. Insofar, our present interpretation of differential amygdala activation awaits empirical replication in future studies employing numbing-specific scales to quantify individual differences.

### Future directions

Previous studies reported a divergence between global emotional responding in an NSSI sample (relative to HC) and lack of altered emotional responses to social exclusion (e.g., Robinson et al., 2023) [51]. The present results show that subgroup analyses can resolve these discrepancies by considering sensitisation to social exclusion and emotional numbing as crucial factors.

To advance individually tailored treatments, future research should investigate the mechanisms involved in emotional numbing, how they interact with psychotherapeutic interventions, and whether preventive interventions could help those with heightened sensitivity to bullying experiences to support functional emotion regulation.

## Supplementary Information

Below is the link to the electronic supplementary material.

Supplementary Material 1 (DOCX 349 KB)

## Data Availability

The data are available from the authors upon request.
