# Supplementary material for: Frequency of bullying modulates amygdala response during social exclusion in participants with non-suicidal self-injury
Source: Eur Child Adolesc Psychiatry. 2026 Jan 16;35(5):1521–31. doi: 10.1007/s00787-025-02940-1 (PMC13272247; doi:10.1007/s00787-025-02940-1)
Supplement: Supplementary file 1 — Supplementary Material 1 (DOCX 349 KB) [file 787_2025_2940_MOESM1_ESM.docx]

**Frequency of bullying modulates amygdala response during social exclusion in participants with non-suicidal self-injury**

**Supplementary Information**

This file contains the following additional information:

- Figure S1: Cyberball experiment
- Investigating putative scanning site effects: Description of procedure and results
- Table S1: Demographic and psychometric measurements with statistical comparisons between healthy controls and participants engaging in non-suicidal self-injury (NSSI)
- Figure S2: Differential brain activation (social exclusion > inclusion) for healthy controls and participants engaging in NSSI


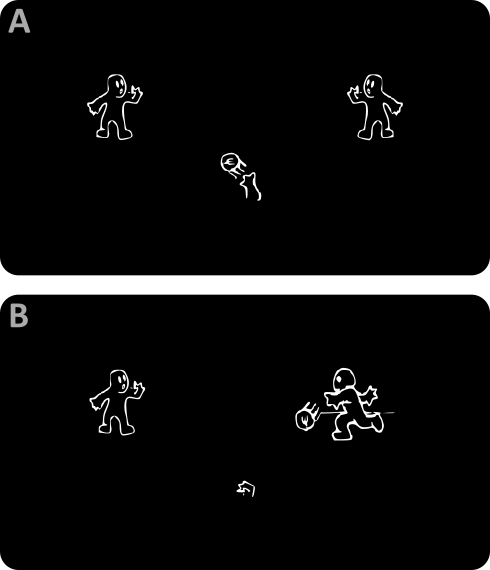


**Figure S1.** Still frames from the Cyberball experiment, illustrating the participant's interaction, represented by the hand at the bottom of the screen. (**A**) captures the moment when the participant throws the ball to the player on the left, and (**B**) depicts the player on the right throwing the ball to the participant. In the “social inclusion” condition, the participant is actively involved in the game. Conversely, in the “social exclusion” condition, the participant, after initial involvement, is deliberately excluded in the later phase of the game. During the “passive watching” condition, the participant is mentally prepared to merely observe the game without any active engagement.

**Investigating putative scanning site effects: Description of procedure and results**

Neuroimaging data were collected at two different scanning sites (Mannheim, Ulm, Germany). The main article presents an SPM12 second-level analysis focusing on the “social exclusion > social inclusion” effect. This analysis did not consider scanning site as a covariate. We omitted “scanning site” from these analyses based on an assessment that evaluated whether this factor influenced the “social exclusion > inclusion” effect before running the analyses presented in the main article: For each participant, the contrast of “exclusion minus inclusion” was calculated. These contrasts were then evaluated with two-sample t-tests within each group of healthy controls and NSSI, and with scanning site as grouping factor. An F-contrast was assessed at a voxel-height threshold of p < 0.001. Neither test indicated significant site differences, even at an uncorrected cluster-level threshold of p < 0.05 for each group. Due to the absence of a scanning site effect, this variable was not further considered, and data were aggregated across scanning sites for the analysis reported in the main article.

**Table S1.** Means ± standard deviations of demographic and psychometric measurements for the healthy control group and the entire group of participants engaging in non-suicidal self-injury (NSSI), with corresponding two-sample t-test statistics. The number of participants differs across variables due to missing data. Abbreviations: BSL-23: Borderline Symptom List-23; CTQ: Childhood Trauma Questionnaire; PHQ-9A: Patient Health Questionnaire-9 for Adolescents

| Variable | Healthy controls | NSSI | t value | p value |
| --- | --- | --- | --- | --- |
| Age | 19.5 ± 2.3  (n = 58) | 19.6 ± 2.1  (n = 57) | t(113) = -0.24 | 0.812 |
| CTQ,  Emotional Abuse | 6.4 ± 2.6  (n = 58) | 16.0 ± 5.4  (n = 48) | t(104) = -11.99 | < 0.001 |
| CTQ,  Physical Abuse | 5.2 ± 0.9  (n = 58) | 7.5 ± 3.3  (n = 48) | t(104) = -5.09 | < 0.001 |
| CTQ,  Sexual Abuse | 5.5 ± 2.1  (n = 58) | 8.6 ± 5.1  (n = 48) | t(104) = -4.19 | < 0.001 |
| CTQ,  Emotional Neglect | 6.5 ± 2.4  (n = 58) | 15.3 ± 5.1  (n = 48) | t(104) = -11.66 | < 0.001 |
| CTQ,  Physical Neglect | 6.3 ± 2.0  (n = 58) | 9.9 ± 4.5  (n = 48) | t(104) = -5.47 | < 0.001 |
| PHQ-9A | 3.4 ± 2.9  (n = 58) | 17.1 ± 5.7  (n = 52) | t(108) = -16.02 | < 0.001 |
| BSL-23 | 5.3 ± 7.8  (n = 57) | 50.2 ± 20.0  (n = 52) | t(107) = -15.72 | < 0.001 |


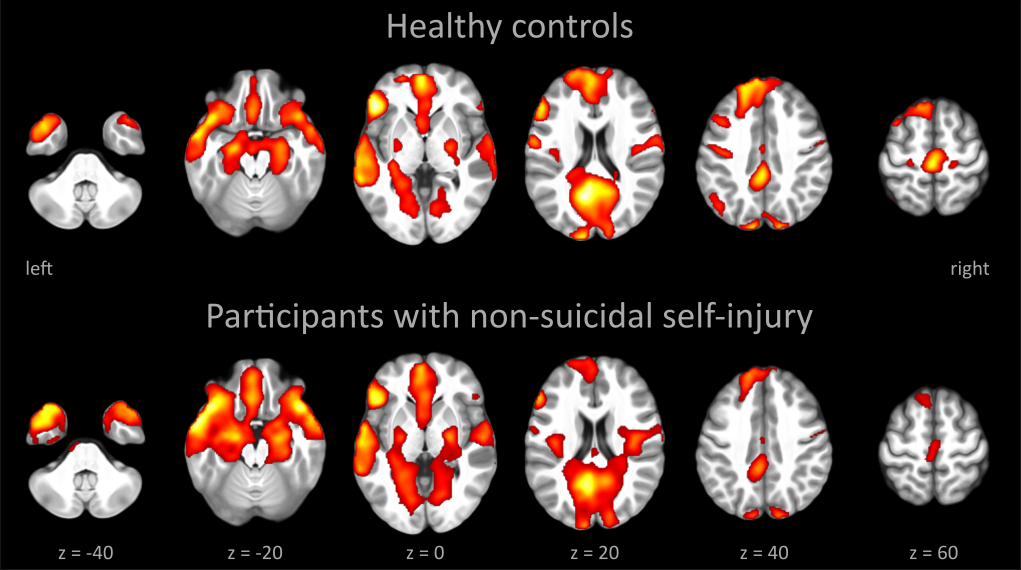


**Figure S2.** Brain activation results for the contrast “social exclusion minus social inclusion”. The upper panel shows the results for healthy controls (n = 58), and the lower panel for participants with non-suicidal self-injury (n = 57). The statistical parametric maps were thresholded at p < 0.001, family-wise error rate-corrected (p < 0.05) at the cluster level (corresponding to a cluster extent threshold of k = 343 voxels). Clusters were overlaid onto the mean normalized skull-stripped T1 image (averaged across all 115 participants), using MRIcroGL (Rorden C, Brett M. Stereotaxic Display of Brain Lesions. Behav Neurol. 2000;12(4):191-200. https://doi.org/10.1155/2000/421719).
